# Structural and functional fractionation of right superior parietal cortex in bistable perception

**DOI:** 10.1016/j.cub.2010.12.009

**Published:** 2011-02-08

**Authors:** Ryota Kanai, David Carmel, Bahador Bahrami, Geraint Rees

**Affiliations:** 1 UCL Institute of Cognitive Neuroscience, University College London, Alexandra House, 17 Queen Square, London WC1N 3AR, UK; 2 Wellcome Trust Centre for Neuroimaging, University College London, London WC1N 3BG, UK; 3 Current address: Department of Psychology and Centre for Neural Science, New York University, 6 Washington Place, New York, NY 10003, USA; 4 Interacting Minds Project, Institute of Anthropology, Archaeology and Linguistics, Aarhus University and Centre of Functionally Integrative Neuroscience, Aarhus University Hospital, Norrebrogade 44, Building 10G, 8000 Aarhus C, Denmark

## Abstract

When faced with ambiguous sensory input, conscious awareness may alternate between the different percepts that are consistent with the input. Visual phenomena leading to such multistable perception, where constant sensory input evokes different conscious percepts, are particularly useful for investigating the processes underlying perceptual awareness [1]. Understanding the role that high-level brain regions outside early visual cortex play in perceptual alternations could elucidate how top-down processes modulate conscious perception [2]. In two studies [3,4] published recently in *Current Biology*, different combinations of the present authors used repetitive transcranial magnetic stimulation (rTMS) to disrupt activity in human superior parietal cortex, and reported seemingly contradictory results [5] concerning the effect of disrupting the normal function of this area on bistable perception. Here we join forces to resolve this discrepancy.

## Main Text

Binocular rivalry occurs when a sufficiently different image is presented to each eye. Rather than being combined into a single percept, the incompatible images compete and perception alternates between each monocular view every few seconds. Carmel *et al.* [3] stimulated a location in the right superior parietal lobule (SPL), where activity is time-locked to perceptual switches in rivalry [6]. Offline disruption of the function of this area shortened dominance durations (increased switch rates) in binocular rivalry compared to no stimulation, whereas stimulating a control site (the homologous locus in the left hemisphere) did not.

**Figure 1:**
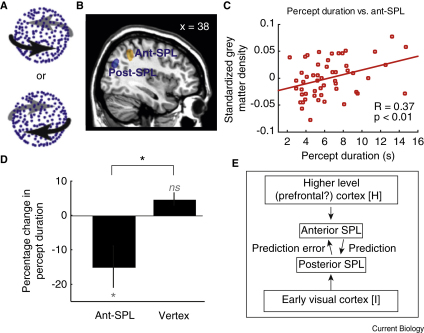
The rotating sphere stimulus, structure/percept correlations, TMS results and a putative mechanism. (A) Schematic depiction of a structure-from-motion rotating sphere. (B) Opposite structure/percept correlations within Right SPL. The posterior blue cluster (x = 38, y = −64, z = 32) depicts a negative correlation between structure-from-motion percept duration and standardized grey matter density at the locus stimulated by Kanai *et al.*[4]. The anterior yellow cluster is reported here for the first time, and depicts a positive correlation in a cluster centred at the locus stimulated by Carmel *et al.*[3] (x = 36, y = −45, z = 51). The clusters are shown at a threshold of t > 2.5 (p < 0.008, uncorrected) for visualization purposes. Post-SPL; posterior SPL; Ant-SPL; anterior SPL. (C) Positive correlation between percept duration and standardized grey matter density in the central voxel of the ROI (x = 36, y = −45, z = 51). Each red circle represents one participant. (D) TMS results. Applying TMS over anterior SPL (but not vertex) significantly decreased percept durations. The baseline in each condition is the mean percept duration before TMS stimulation. ^∗^p < 0.05 (n = 8); grey text and asterisks represent comparisons with zero; the black asterisk represents a comparison between conditions. (E) Applying hierarchical Bayesian network theory to bistable perception. Sensory input [I] enters the system through early visual cortex. Higher-level regions generate hypotheses [H] about the environment, leading to predictions about how to interpret the input, and prediction error that reflects the difference between the prediction and input. The present findings suggest that predictions may be generated in anterior SPL, whereas prediction error mechanisms may reside in posterior SPL.

A different kind of bistability arises in structure-from-motion perception. For example, a field of moving dots can be seen as a sphere that rotates clockwise or counterclockwise (Figure 1A). Here too, the different interpretations compete for dominance, alternating every few seconds. Kanai *et al.* [4] applied rTMS to a more posterior locus than Carmel *et al.* [3] within SPL. The choice of this location was based on the finding that grey matter density in this location predicted percept duration for a bistable rotating sphere. Kanai *et al.* [4] found an opposite effect of TMS to Carmel *et al.* [3]: offline disruption of SPL activity (bilaterally) increased percept durations (decreased switch rates) for these bistable stimuli compared to no stimulation, whereas stimulation of a control area (the vertex) did not.

To resolve this apparent contradiction, we first revisited the brain-structure/percept-duration correlation reported by Kanai *et al.* [4]. The regions stimulated in that study were the only ones for which the statistical significance of the reported correlations survived a family-wise error correction for multiple comparisons. We reasoned that the findings of Carmel *et al.* [3], as well as the previous neuroimaging results [6], now justified a more sensitive region-of-interest (ROI) approach to interrogate the relationship between the structure of anterior SPL and bistable perception. Indeed, this ROI analysis revealed a positive correlation (R = 0.37, t(50) = 2.78, p < 0.01) — the opposite sign to that found more posteriorly by Kanai *et al.* [4] — between grey matter density and rotating sphere switch rate (Figure 1B,C). Furthermore, this positive correlation was lateralized to the right SPL (R = 0.03, t(50) = 0.28, p = 0.78 at the left-hemisphere coordinate, x = −36, y = −45, z = 51), in line with Carmel *et al.*'s [3] findings for rivalry (see Supplemental Information for full methods and analyses).

Having established that the structure of posterior [4] and anterior right SPL show opposite relations to the dynamics of bistable perception, we next hypothesized that the anterior region may play the same functional, causal role in structure-from-motion perception as it did in rivalry. To test this prediction, we applied rTMS to this location (see Supplemental Information for details of rTMS methodology and experimental protocol) just before eight healthy volunteers viewed the same bistable, structure-from-motion stimulus used previously by Kanai *et al.* [4]. Percept durations for the rotating sphere decreased (as had happened with binocular rivalry [3], and in contrast to when rTMS was applied to posterior SPL [4]). Stimulation of a control site (vertex) had no effect (Figure 1D).

For the same bistable stimulus, disruption of posterior SPL slowed perceptual switching [4], whereas disruption of anterior SPL made it faster. These results clarify that the seemingly discrepant previous findings [3,4] are unlikely to be due to different neural bases for different forms of bistability, nor to dissimilar stimulation protocols [5]. Rather, they reflect a fractionation of parietal cortex function, such that different regions within parietal cortex play opposing roles in the control of bistability.

These findings imply that further research on separate functions residing within the SPL is required in order to develop an understanding of visual awareness. One promising theoretical approach to guide such research may be found in hierarchical Bayesian network theory [7], which suggests that sensory input is initially processed in early visual cortex, while higher-level brain regions seek to infer the most likely environmental cause that gave rise to the input by generating forward models or hypotheses. These hypotheses are top-down predictions that are fed back to lower level sensory areas to explain the bottom-up sensory signal. The discrepancy between the top-down prediction and the bottom-up input is the prediction error — the remaining, unexplained portion of the bottom-up signal. Top-down predictions are updated in subsequent iterations of this recurrent process to further minimise the prediction error. In the case of bistable stimuli, this dynamic process is perpetual: though the input remains the same, the difference between the present interpretation and the alternative one exerts constant pressure to alter the current hypothesis, and this happens periodically. By this account, anterior SPL may be involved in generating the prediction (which corresponds to the current interpretation); impairing its activity would thus lead to a weaker and more changeable interpretation (and faster perceptual switching). Conversely, posterior SPL may be involved in generating the prediction error signal, which would increase the probability of a perceptual switch; impairing its activity would thus lead to slower switches (Figure 1E).

The present findings resolve an intriguing contradiction [5]: whether right SPL activity makes the perceptual alternations of a bistable stimulus faster or slower depends on the precise location within this region that one looks. The balanced activity of different neural substrates residing in this area may underlie a host of functions related to the generation of conscious perception. A better understanding of this fractionation therefore represents a worthy pursuit in the ongoing attempt to understand consciousness.

Finally, a further twist to the story comes from a recent study by Zaretskaya *et al.* [8]. Contrary to our findings, they reported that online TMS over an area very close to our right anterior SPL decreased the switch rate for binocular rivalry. Although the impact of TMS on neural activity is most parsimoniously explained as injection of noise [9], online versus offline application of TMS may affect neural activity differently, leading to different effects on bistability dynamics [8]. Importantly, our present study compared the effects of TMS at different anatomical loci using the identical offline TMS protocol and the stimulus as one of our previous studies [4]. Thus, the fractionation reported here can be safely attributed to differences in the roles of anterior and posterior rSPL.

## Supplemental Information

Document S1. Supplemental Experimental Procedures, Supplemental Results, Supplemental References and two figure

## References

[1] Blake R., Logothetis N.K. (2002). Visual competition. Nat. Rev. Neurosci..

[2] Rees G., Kreiman G., Koch C. (2002). Neural correlates of consciousness in humans. Nat. Rev. Neurosci..

[3] Carmel D., Walsh V., Lavie N., Rees G. (2010). Right parietal TMS shortens dominance durations in binocular rivalry. Curr. Biol..

[4] Kanai R., Bahrami B., Rees G. (2010). Human parietal cortex structure predicts individual differences in perceptual rivalry. Curr. Biol..

[5] Clifford C.W.G. (2010). Visual perception: Ambiguity involving parietal cortex. Curr. Biol..

[6] Lumer E.D., Friston K.J., Rees G. (1998). Neural correlates of perceptual rivalry in the human brain. Science.

[7] Friston K. (2008). Hierarchical models in the brain. PLoS Comput. Biol..

[8] Zaretskaya N., Thielscher A., Logothetis N.K., Bartels A. (2010). Disrupting parietal function prolongs dominance durations in binocular rivalry. Curr. Biol..

[9] Schwarzkopf D.S., Silvanto J., Rees G. (2011). Stochastic resonance effects reveal the neural mechanisms of transcranial magnetic stimulation. J. Neurosci..

