# Supplementary material for: Structural and functional fractionation of right superior parietal cortex in bistable perception
Source: Curr Biol. 2011 Feb 8;21(3):R106–7. doi: 10.1016/j.cub.2010.12.009 (PMC3084447; doi:10.1016/j.cub.2010.12.009)
Supplement: Document S1. Supplemental Experimental Procedures, Supplemental Results, Supplemental References and two figure [file mmc1.pdf]

Supplemental Information

Structural and functional fractionation of right superior parietal cortex in bistable perception

Ryota Kanai, David Carmel, Bahador Bahrami and Geraint Rees

Supplemental Experimental Procedures

Voxel-based morphometry

The data used for the voxel-based morphometry (VBM) analysis were identical to those described in Kanai et al. (S1). A total of 52 healthy volunteers with normal or corrected to normal vision (ages 19–38, mean 25.8 ± 5.14 S.D.; 29 female) were recruited. Written informed consent was obtained from all participants before data collection. The local ethics committee approved the experiments.

As in our previous study (S1), effects of age and gender were regressed out as covariates of no interest. For the ROI analysis, the standardized grey matter density was extracted for the coordinate (x = 36, y = -45, z = 51) reported in Carmel et al. (S2).

TMS experiment

The procedure used for the TMS experiment was identical to that described in Kanai et al (S1), and was approved by the local ethics committee. Eight healthy participants (4 females, age = 21.4 ± 1.9 s.d. years old) with normal or corrected-to-normal vision gave written informed consent to participate in two TMS sessions. In each session, participants completed three 4-minute blocks reporting their ambiguous motion percepts before TMS stimulation, and another 3 blocks immediately after. The theta-burst TMS stimulation protocol consisted of 3 pulses at 50Hz repeated at 200ms intervals for 40 seconds at 40% stimulator output (S3). The mean pre-TMS percept duration was calculated based on the second and third blocks. The mean post-TMS percept duration was calculated from all the 3 blocks. The two stimulation sites (anterior SPL and vertex) were tested on separate days and the order of the sites was counterbalanced across participants.

Supplemental Results

Statistical results of VBM

In the main text, we reported the correlation between individuals' percept duration and grey matter density at the coordinate reported in Carmel et al. (S2) (x = 36, y = -45, z = 51). A similar positive correlation between percept duration and grey matter density was also revealed when a small volume correction (SVC) was applied for a region including this coordinate (sphere radius = 15mm; sphere centre x = 36, y = -45, z = 51; MNI coordinate of the peak voxel within the sphere, x = 42, y =-48, z = 40; $p_{FWE\text{-}corr} = 0.01$; Z=3.48), providing consistent results.

The positive correlation between anterior SPL and percept duration did not reach

statistical significance when corrected for multiple comparisons across the whole brain (S1). However, when a cluster level analysis with an initial threshold of $p < 0.001$ (uncorrected) was applied, the size of the significant cluster in anterior SPL was 445.5mm^3 (132 voxels, 1.5mm^3 each, i.e., 3.375mm^3 per voxel). When corrected for the whole brain, statistical significance at the cluster level was p=0.11, but the peak voxel showed a strong correlation ($R = 0.48$, $p < 0.001$). Thus, the null finding at a whole-brain corrected level could be due to lack of statistical power (i.e., an insufficient number of data points) to detect the positive correlation in anterior SPL.

Structural relationship between SPL regions

To determine the relationship of grey matter density between the parietal regions identified by VBM analysis, we took grey matter density from the peak voxels in each region that showed the most significant correlation with percept duration (voxels in right pSPL and left SPL were those identified previously by Kanai et al. (S1)). The correlation between those regions was examined by partial correlation with contributions of age, gender and total gray matter volume regressed out.

The results are summarized in Fig. S1. There was a positive correlation between Left SPL and right pSPL across participants ($R = 0.36$, $p < 0.05$). These areas were previously shown to negatively correlate with percept duration (S1). Right anterior SPL was negatively correlated with right posterior SPL, though this relation was only marginally significant ($R = 0.25$, $p = 0.08$), suggesting that common developmental factors such as genetic or environmental contributions may underlie these regions' structural organization. Our present TMS results, together with our previous TMS findings (S1) indicate that these areas independently contribute to an individual's switch rate, because disruption of any of these regions had significant impact on their switch rate.

Supplemental Figure 1

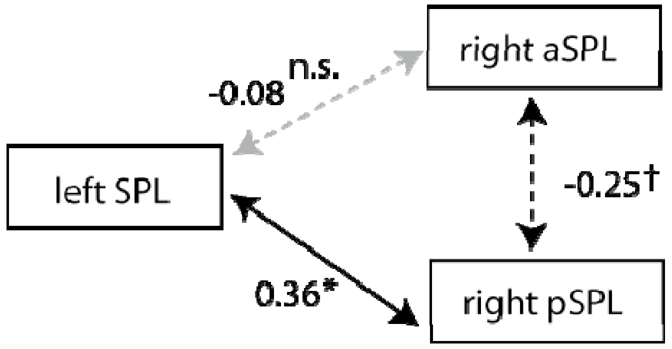

Fig. S1. Inter-regional structural corrections among right anterior SPL (aSPL), right posterior SPL (pSPL) and left SPL. Partial correlations between the grey matter density of the peak coordinates from the three clusters are summarized while regressing out factors of age, gender and total grey matter volume. * $p < 0.05$, † $p = 0.08$, n.s. $p = 0.59$.

Supplemental References

S1. Kanai, R., Bahrami, B., and Rees, G. (2010). Human parietal cortex structure predicts individual differences in perceptual rivalry. Curr. Biol. 20, 1626–1630.

S2. Carmel, D., Walsh, V., Lavie, N., and Rees, G. (2010). Right parietal TMS shortens dominance durations in binocular rivalry. Curr. Biol. 20, R799–R800.

S3. Huang, Y.-Z., Edwards, M.J., Rounis, E., Bhatia, K.P., and Rothwell, J.C. (2005). Theta burst stimulation of the human motor cortex. Neuron 45, 201-206.
